# Towards a Metropolitan Fundamental Diagram Using Travel Survey Data

**DOI:** 10.1371/journal.pone.0148660

**Published:** 2016-02-11

**Authors:** Kai Wang, David M. Levinson

**Affiliations:** 1 Beijing Key Laboratory of Traffic Engineering, Beijing University of Technology, Beijing, China 100124; 2 Department of Civil, Environmental, and Geo- Engineering, 500 Pillsbury Drive SE, Minneapolis, MN 55455, United States of America; Beihang University, CHINA

## Abstract

Using travel diary data from 2000–2001 and 2010–2012 this research examines fundamental traffic relationships at the metropolitan level. The results of this paper can help to explain the causes of some traffic phenomena. Network average speed by time of day can be explained by trip length and cumulative number of vehicles on the road. A clockwise hysteresis loop is found in the Metropolitan Fundamental Diagram in the morning period and a reverse process happens in the afternoon.

## Introduction

Scientific analysis of traffic and travel behavior are related fields that have seen little intersection, owing to their different histories, data sources, and methods. Traditionally travel diaries provide data about a sample of individual travelers, and serving as the data source for estimating regional travel demand models, while traffic measurement gauges network performance. Travel surveys are generally weighted to develop numbers for the region as a whole. Thus large diaries also implicitly contain aggregate data that can be exploited to better understand the causes of variation in traffic conditions. This study explores one region’s travel surveys from two points in time using tools that have historically been used with traffic analysis. It tests some of the relationships that have been uncovered by analysis of data from traffic sensors, to see whether they hold at the metropolitan level.

To date analyses of fundamental relationships of traffic have used traffic data from place-based sensors like loop detectors or GPS. Some representative studies related to network flows relationships, and the macroscopic fundamental diagram (MFD) in particular, are described below.

Network level traffic flow relationships have been periodically considered in the literature. Based on a kinetic theory of traffic, a two-fluid model for the evolution of speed distribution on highways has been proposed [1]. A simulation model with embedded macroscopic rules shows the dynamic behavior of relative concentrations and traffic flows on the boundary and interior links, such that average speed decreases as network concentration increases [2]. The macroscopic traffic network relationship held for road sections, and varies with number of lanes and block length [3]. Large scale simulations reproduced the relationships on larger urban networks [4].

A macroscopic fundamental diagram (MFD) linking space-mean flow, density and speed for a complete network of a large urban area has been demonstrated with a field experiment in Yokohama (Japan)[5]. The data is collected by a combination of fixed detectors and floating vehicle probes (GPS-equipped taxis). The estimates of space-mean speeds and densities at different times of day lie close to a smoothly declining curve with deviations smaller than those of individual links. The spatial distribution of vehicle density in the network is one of the key components of a low scatter MFD and its shape [6]. If the spatial distribution of link density is the same for two different time intervals with the same number of vehicles in the network, then the same average flows should be presented. They also examine the errors of an MFD based on the errors of individual link microscopic fundamental diagrams (*μ*FD) and errors in the probability density function of link occupancy. Full information of trajectories from probe vehicles for estimating the network speed is superior to spot sensors like loop detectors [7].

Hysteresis was identified on Minnesota freeways, which also have a high scatter due to different spatial and temporal distributions of congestion for the same level of average density [8]. Another reason is synchronized occurrence of transient periods and capacity drop phenomena in the offset of congestion. Similarly, a clockwise hysteresis loop exists in MFD [9]. The research also indicates that drivers tend to choose routes adaptively to avoid congested areas. Speeds subsequently recover from congestion, so the presence of adaptive routes make hysteresis less likely. To explore the limiting properties of network-wide traffic flow relations under congestion conditions, a model reproducing hysteresis and gridlock of the urban street network was established [10]. It is found that the network flow can be approximated as a non-linear function of network average density and variation in link densities, the networks with multiple route options tend to congest at a smaller density range. Traffic demand management and adaptive driving affect gridlock size, propagation speed, and recovery speed.

The impact of heterogeneity on the existence of an MFD with data collected by loop detectors has been studied [11]. Also a hysteresis pattern was observed which may be caused by spatially heterogeneous nature of travel demand, including different schedules for cars and trucks. The spatial distribution of congestion affects the scatter and hysteresis of MFD [12].

We note that the MFD suffers from the Modifiable Areal Unit Problem (MAUP), which means the size and precise shape of the MFD will be affected by the size of the research area. The type of roads and the way of dividing the city into zones can impact the size and shape of the MFD.

Average vehicle densities can be estimated across a network by using travel speed and MFD of urban traffic [9, 13]. Congestion is critically important to the existence of MFD, and the estimates based on average travel speed are inaccurate in non-congested states but reliable and accurate in congestion or its onset. Average trip distance increases with network load level, as congestion induces drivers to take longer routes [14].

In contrast with the existing research described above, this paper employs travel diaries from a metropolitan survey to examine questions that have heretofore used traffic data. Using travel surveys conducted by the Metropolitan Council of the Minneapolis/Saint Paul region during 2010–2012 as well as 2000–2001, this paper aims to develop a fundamental diagram that relates speed to the total number of vehicles on the network at a given time, and to estimate a statistical relationship that accounts for that, trip length, and the onset / offset of congestion.

## Materials and Methods

### Data

The 2000 and 2010 Twin Cities Travel Behavior Inventory (TBI) (with data collection in 2000–2001 and 2010–2012 respectively) are used in this analysis, the two time periods allow tests of temporal stability. The data, summarized in [15], represent the entire survey period. All data were collected on weekdays, and each trip record in the data provides location of origins and destinations, departure time, mode, and purpose. The spatial coverage of trips is the seven-county metropolitan region (Anoka, Carver, Dakota, Hennepin, Ramsey, Scott, and Washington counties in Minnesota).

We examine only trips by automobile in this analysis, though the survey was multi-modal. Future research may investigate MFDs for other or all modes of travel. The travel duration was reported by survey respondents. A consultant provided household weights for the survey as described in [16]. The research team computed network distances for each trip in the survey, assuming trips took the shortest distance path on the network, using the region’s TLG (Lawrence Group) network.

Much of the data is self-reported by the individuals who participated, and therefore there are errors in reporting. The following censoring rules were employed to address this issue, as illustrated in Table 1. Trips were excluded if:

- The calculated travel speed was higher than 120km/h or lower than 10km/h.
- The reported travel time was higher than 120 mins or lower than 1 min.
- Weight of trip equals to zero. (The total of all person weights in the weighted survey equals the population of the survey region. If the weight of a trip is given as zero this means the individual is oversampled or there is some other data problem, so it is excluded.)
- Trips began earlier than 3 am or ended after midnight.

**Table 1:** Summary Statistics of TBI (2000 and 2010).

	2000	2010
Number of Households	4860	8195
Number of People	8806	14432
Total Weight	2642056	2849567
Max Household Size	8	8
Max Trips per Person	35	11
Mean Trip Duration (hr)	0.306	0.325
Mean Trip Distance (km)	10.655	8.123
Filters		
Number of Initial Trip Records	56811	115821
- Number of Trip Records Out of Region	9867	12384
- Number of Trip Records Lack Region Information	535	3075
- Number of Trip Records Without Weight	0	31537
- Number of Non-Car Trips	4346	9233
- Number of Trip Records Speed < 10 (km/h)	10064	8665
- Number of Trip Records Speed > 120 (km/h)	986	3202
- Number of Trip Records Duration < 1 (min)	0	0
- Number of Trip Records Duration > 120 (min)	21	40
Number of Trip Records Used in the Research	30992	47685

In addition there are biases. A subsample of the subjects in the TBI2010 survey (too small to use for these purposes) were tracked using an on-person GPS unit for 7 days. For those respondents we do have the actual route chosen [17]. Using different data the median value of the actual path for commute trips is about 30 percent longer than the shortest network path travel time estimated using GPS data [17, 18].

Further, people exaggerate their travel times (which is reported in travel surveys as higher than actual) by on average 50 percent, though this bias depends on network structure and congestion levels and the nature of the trip [19]. Some of this may be due to definitions about when and where a trip begins (e.g. vehicle based GPS will measure engine-on to engine-off, while a subject might report door-to-door travel times).

$$\begin{array}{c} s_{calculated}=l_{shortestpath}/t_{reported} \end{array}$$ (1)

$$\begin{array}{c} s_{adjusted}=\left(l_{shortestpath}*1.3\right)/\left(t_{reported}/1.5\right) \end{array}$$ (2)

Which means actual speeds may be 1.3*1.5 = 1.95 times faster than calculated, depending on assumptions and actual values.

This paper uses reported travel times and estimated shortest network distance paths and reports *s*_*calculated*_, but this bias should be kept in mind. We do not believe it affects the general findings and fundamental relationships, though clearly affects the values associated with those findings. Future research using GPS recordings rather than self-reporting of travel surveys should improve the accuracy and precision of these analyses. Presently the sample sizes of GPS surveys are too small to draw sufficiently general conclusions.

### Nomenclature

Variable: Definition

 index *i*: Trip ID

 index *h*: Household ID

 index *t*: Time of day, time window beginning at a given time of day

 index *full*, *part*, *non*: Trip purpose: Full time work, Part time work, Non work.

 *A*(*t*): Cumulative arrivals

 *a*(*t*): Arrival rate in time interval *t*

 *c*: indicates onset time of congestion [1, 0]

 *D*(*t*): Cumulative departures

 *d*(*t*): Departure rate in time interval *t*

 *L*(*t*): Average travel distance of trips with start time located in time interval beginning at *t*

${\bar{L}}_{i}$: *Average travel distance of all trips*

 *l*_*i*_ : Travel distance of trip *i*

 *N*(*t*): Number of vehicles on the network, it is the difference of *A*(*t*) − *D*(*t*)

 *P*: Regional Population

 *p*_*h*_: Number of people in household *h*

 *S*(*t*): Average speed of trips with start time located in time interval beginning at *t*

${\bar{S}}_{i}$: *Average trip speed of all trips*

 *s*_*i*_: Speed of trip *i*

 *s*_*calculated*_: Calculated speed by shortest path distance over reported trip duration.

 *s*_*adjusted*_: Adjusted value of *s*_*calculated*_

 *t*_*a*_(*i*), *t*_*d*_(*i*): The start time and end time of the trip *i*, travel time of trip *i* is *t*_*i*_ = *t*_*d*_(*i*) − *t*_*a*_(*i*).

 *t*_*i*_: Travel duration for trip *i*

 *T*(*t*): Average of trip duration in time interval *t*

 *T*(*i*): Total travel time of all trips

$\bar{T}\left(i\right)$: The average travel time of all trips

 *w*_*h*_: Weight of household *h*

 *w*_*p*,*h*_: Unadjusted Person weight of individuals in household *h*

 *w*_*i*_: Adjusted person and trip weight

 λ: Weight adjustment rate

 *X*_*a*,*i*_ (*X*_*d*,*i*_): Indicating whether the start time (end time) of trip *i* is located in time interval *t*

 *y*: Duration of time window beginning at *t*

### Definitions

For every trip, the start time and end time of travel was reported. This allows calculation of the cumulative number of vehicles entering and exiting the network, based on summing data from individual trips by time of day.

The cumulative number of arrivals (trips entering the network) by time *t* (*A*(*t*)) is the sum of arrivals in each time period (*a*(*t*)) (running from minute 180 (3 am) until 1440 (midnight)):

$$\begin{array}{c} A\left(t\right)=\sum_{t=180}^{1440}a(t) \end{array}$$ (3)

Travel surveys weight individuals to try to replicate socio-economic and demographic distributions in the general population. So for a 1 percent sample, the average person weight would be 100. Oversampled groups would receive lower weights, while undersampled groups receive a higher weight.

In TBI surveys the simple rate is presented by a household weight *w*_*h*_ developed by the consultants who conducted the survey to achieve the above aims. In this paper, we require a person weight *w*_*p*,*h*_ in household *h* for every person in the household, which is obtained by equally distributing the household weight to the members of the household.

$$\begin{array}{c} w_{p,h}=\frac{w_{h}}{p_{h}} \end{array}$$ (4)

where: *p*_*h*_ is the number of people in household *h*.

The sum of *w*_*p*,*h*_ for all respondents should be the population size (*P*) of the seven-county region. While the initial weights aimed to achieve that as well, our application of filters would result in the weights needing adjustment to reacquire that total.

$$\begin{array}{c} P=\lambda *\sum_{h=1}^{H}w_{h}=\lambda *\sum_{h=1}^{H}w_{p,h}*p_{h} \end{array}$$ (5)

$$\begin{array}{c} w_{i}=\lambda *w_{p,h} \end{array}$$ (6)

where: λ is the coefficient of weight adjustment. *w*_*i*_ is the person weight. (The person weight equals the trip rate as a person can only be on one trip at a time).

The arrivals in each time window of duration *y* are the sum of all trips starting at *t* multiplied by the weight of the trip (*w*_*i*_).

$$\begin{array}{c} a\left(t\right)=\sum_{i=1}^{I}X_{a,i}\left(t\right)*w_{i} \end{array}$$ (7)

where: *X*_*a*,*i*_(*t*) = 1 if *t* ≤ *t*_*a*_(*i*) < *t* + *y*, that is, if trip *i* originates in the time window of duration *y* indexed by *t*, and 0 otherwise. The subscript *i* indexes trips, *I* is the total number of trips.

Similarly, the cumulative number of departures (trips leaving the network) by time *t* (*D*(*t*)) are the sum of individual departures in each time period (*d*(*t*)):

$$\begin{array}{c} D\left(t\right)=\sum_{t=180}^{1440}d(t) \end{array}$$ (8)

The time at which a trip leaves is simply the time in which it enters plus the duration of the trip (its length divided by speed).

$$\begin{array}{c} t_{d}\left(i\right)=t_{a}\left(i\right)+\frac{l_{i}}{s_{i}} \end{array}$$ (9)

The departures in each time period are the sum of all trips ending in a time window of duration *y* beginning at *t* multiplied by the weight of the trip (*w*_*i*_).

$$\begin{array}{c} d\left(t\right)=\sum_{i=1}^{I}X_{d,i}\left(t\right)*w_{i} \end{array}$$ (10)

where *X*_*d*,*i*_(*t*) = 1 if *t* ≤ *t*_*d*_(*i*) < *t* + *y*, meaning trip *i* leaves the network in the time window, and 0 otherwise.

The total number of vehicles on the road at any given time *N*(*t*) is the difference between cumulative arrivals and cumulative departures.

$$\begin{array}{c} N(t)=A(t)-D(t) \end{array}$$ (11)

The travel duration of each trip is given by:

$$\begin{array}{c} t_{i}=t_{d}\left(i\right)-t_{a}\left(i\right)=\frac{l_{i}}{s_{i}} \end{array}$$ (12)

Total travel duration (*T*(*i*)) is thus the sum of this over all trips:

$$\begin{array}{c} T\left(i\right)=\sum_{i=1}^{I}\frac{l_{i}}{s_{i}} \end{array}$$ (13)

Average travel duration is

$$\begin{array}{c} \bar{T}\left(i\right)=\frac{T(i)}{I}=\frac{1}{I}\sum_{i=1}^{I}\frac{l_{i}}{s_{i}}=\sum_{i=1}^{I}\frac{l_{i}/I}{s_{i}/I}\neq \frac{\sum_{i=1}^{I}l_{i}/I}{\sum_{i=1}^{I}s_{i}/I}=\frac{{\bar{L}}_{i}}{{\bar{S}}_{i}} \end{array}$$ (14)

In short, we cannot use network average lengths and speeds to correctly ascertain average travel duration (though for large samples, they may be similar), instead we construct the average from individual trip records.

## Results

### Descriptive Results

**Fig 1:**
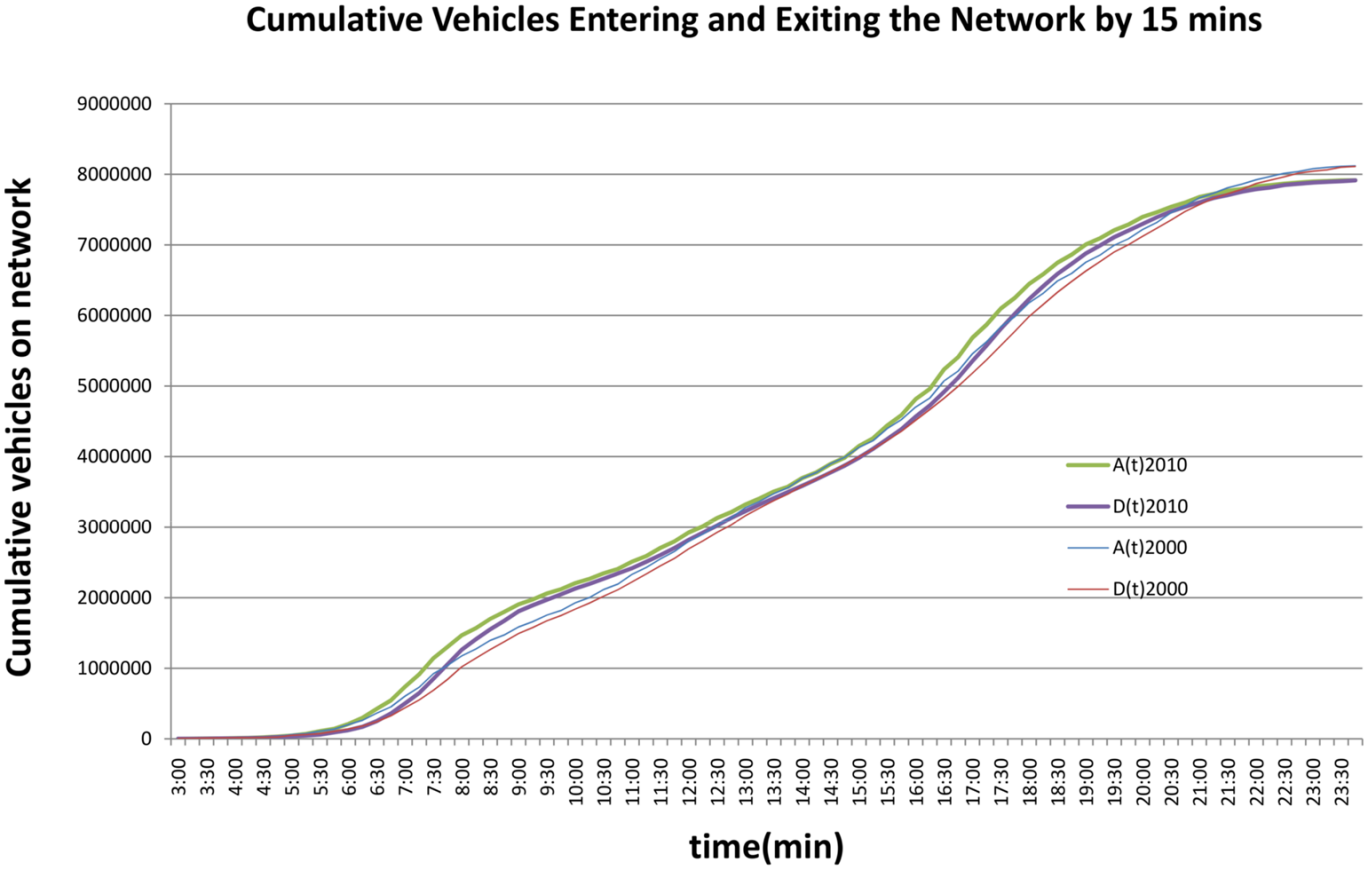
Cumulative Arrivals and Departures Curve (2000 and 2010).

Fig 1 compares *A*(*t*) with *D*(*t*), the cumulative arrivals and departures across the day. Interestingly, total trips in 2010 and 2000 were approximately equal (despite an about 10 percent increase in regional population), consistent with observations of peak travel [20–22]. Moreover the number of trips in 2000 were higher in mid-day than 2010, though at the end of the morning peak, the numbers had been similar. This supports observations of a decline in off-peak (typically non-work) travel between these two periods.

**Fig 2:**
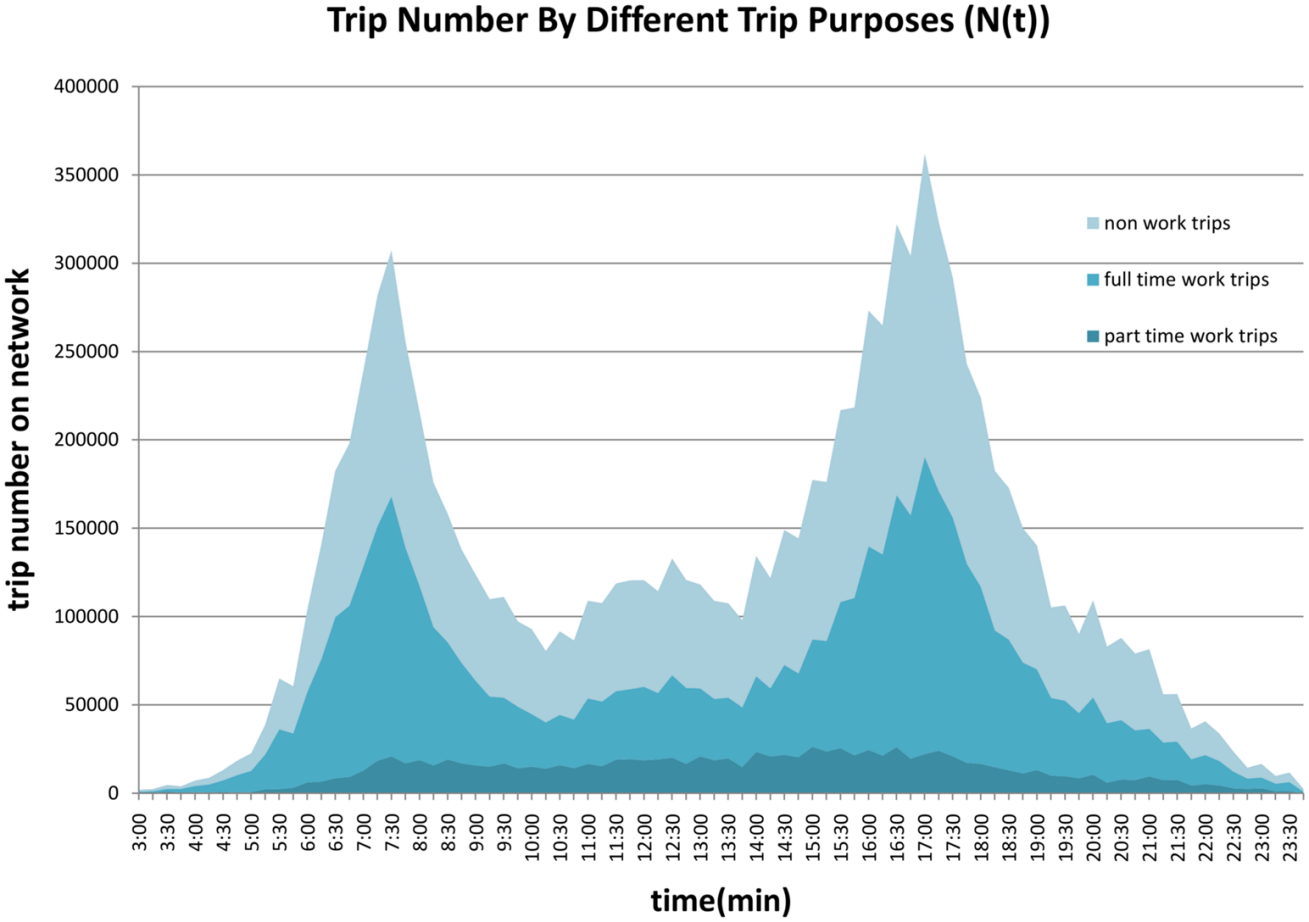
Number of Trips on the Network by Purpose (2010).

Next the change of the total number of on-road vehicles by time of day, *N*(*t*) is presented in Fig 2, which graphs the weighted diurnal curves for the metropolitan region. There are two spikes at around 7 am and 5 pm. Slopes of the line indicate the onset and offset of congestion, illustrating the rush hours in the morning and afternoon periods. The slopes indicate change in *N*(*t*) by time of day. With travel surveys, unlike traffic data, we know the purpose of the trip, so we can disaggregate travel by purpose. Travel behaviors (such as trip length and time-of-day) and thus congestion experienced vary by purpose. Fig 2 compares full and part-time workers and non-workers (*N*_*full*_(*t*), *N*_*part*_(*t*), *N*_*non*_(*t*)). First we see there are many more full time workers than part-time workers. Second we see that part-time workers are much more likely to be on the road in the middle of the day, while full-time workers are more peaked in their travel patterns. This peaked nature of (full-time) work trips gives rise to congestion. The figure also shows that non-work trips are more common in the afternoon. While the number of non-work trips is far higher than the number of work trips, the greater length of work trips offsets that to some extent, so during the peaks most vehicles on the road are work trips, even if most trips that originate in that period are not.

**Fig 3:**
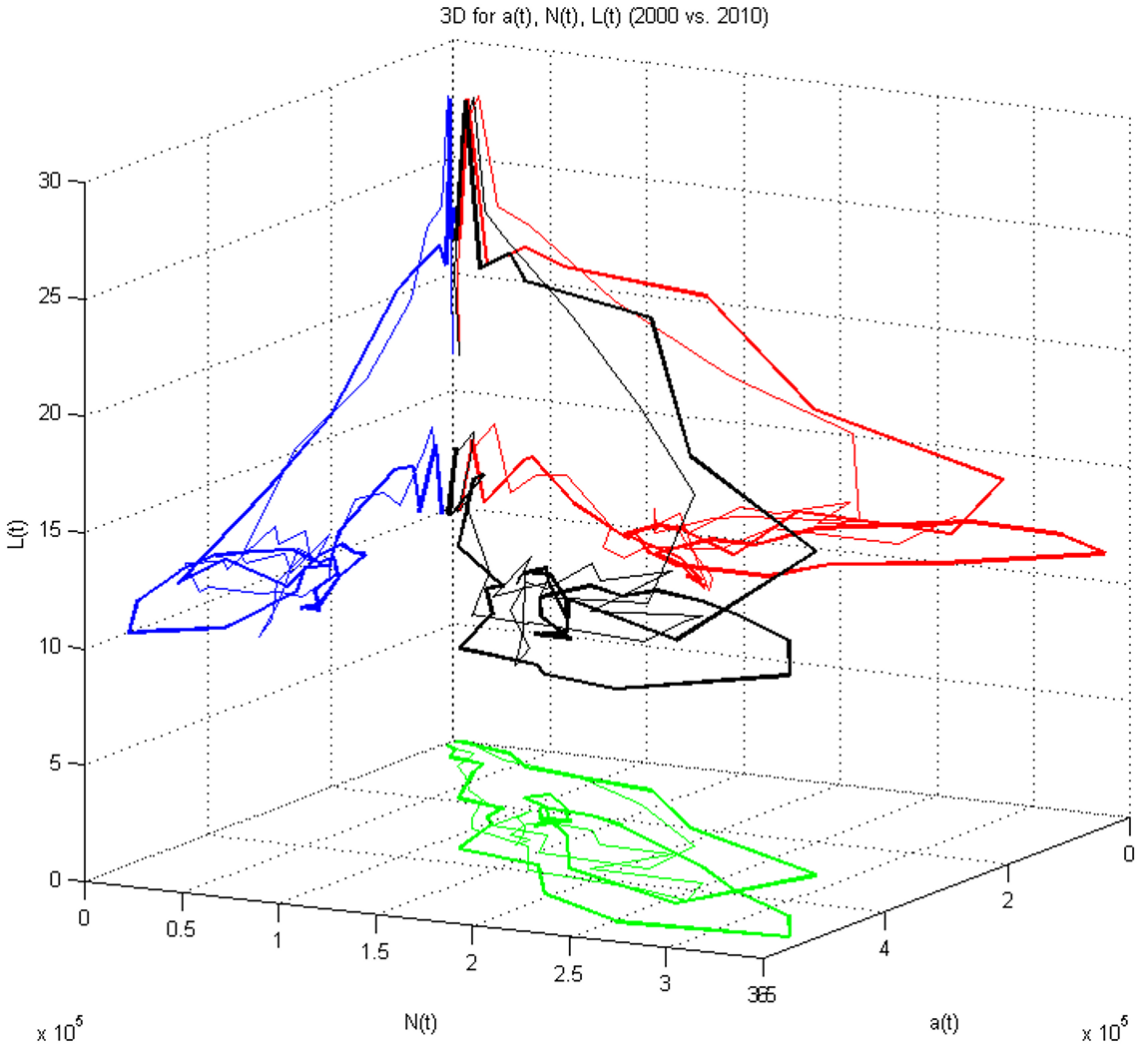
3D Relationship Among a(t), N(t) and L(t) (2000 and 2010).

**Fig 4:**
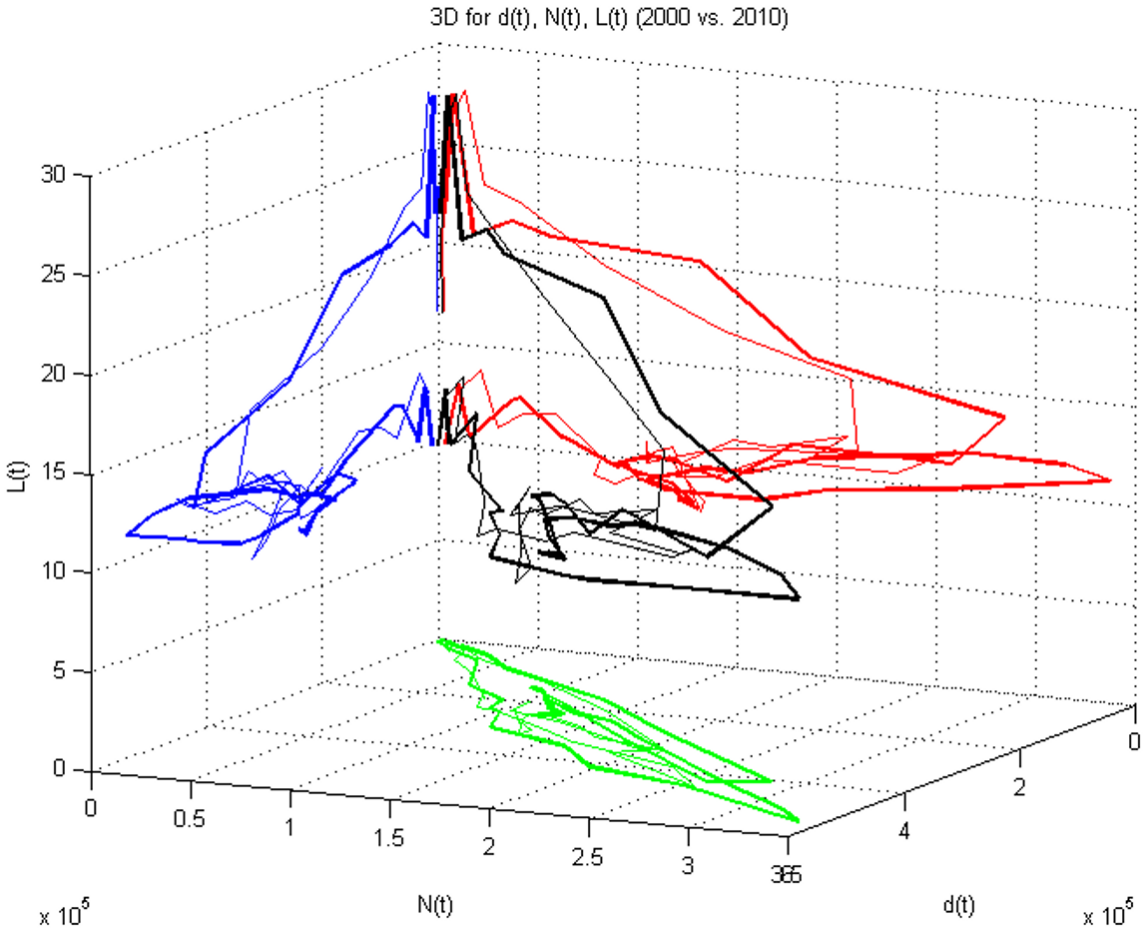
3D Relationship Among d(t), N(t) and L(t) (2000 and 2010).

Figs 3 and 4 shows the relationships between arrivals (departures), number of vehicles on the network, and trip length in three dimensions (thin lines represent 2000, thick lines 2010). In traditional microscopic fundamental diagrams, length is fixed. Even in the MFD, the distances are usually fixed. But this figure allows exploration of average trip length by 15 minute period explicitly. Length is clearly higher in the off-peak periods, and drops as total travel increases. The most common trip length is between 18 and 20 km. The blue line relates *L*(*t*) and *a*(*t*), the red line relates *L*(*t*) and *N*(*t*), and the green line relates *N*(*t*) and *a*(*t*), while the black line shows the three dimensional relationship for *a*(*t*), *N*(*t*) and *L*(*t*).

**Fig 5:**
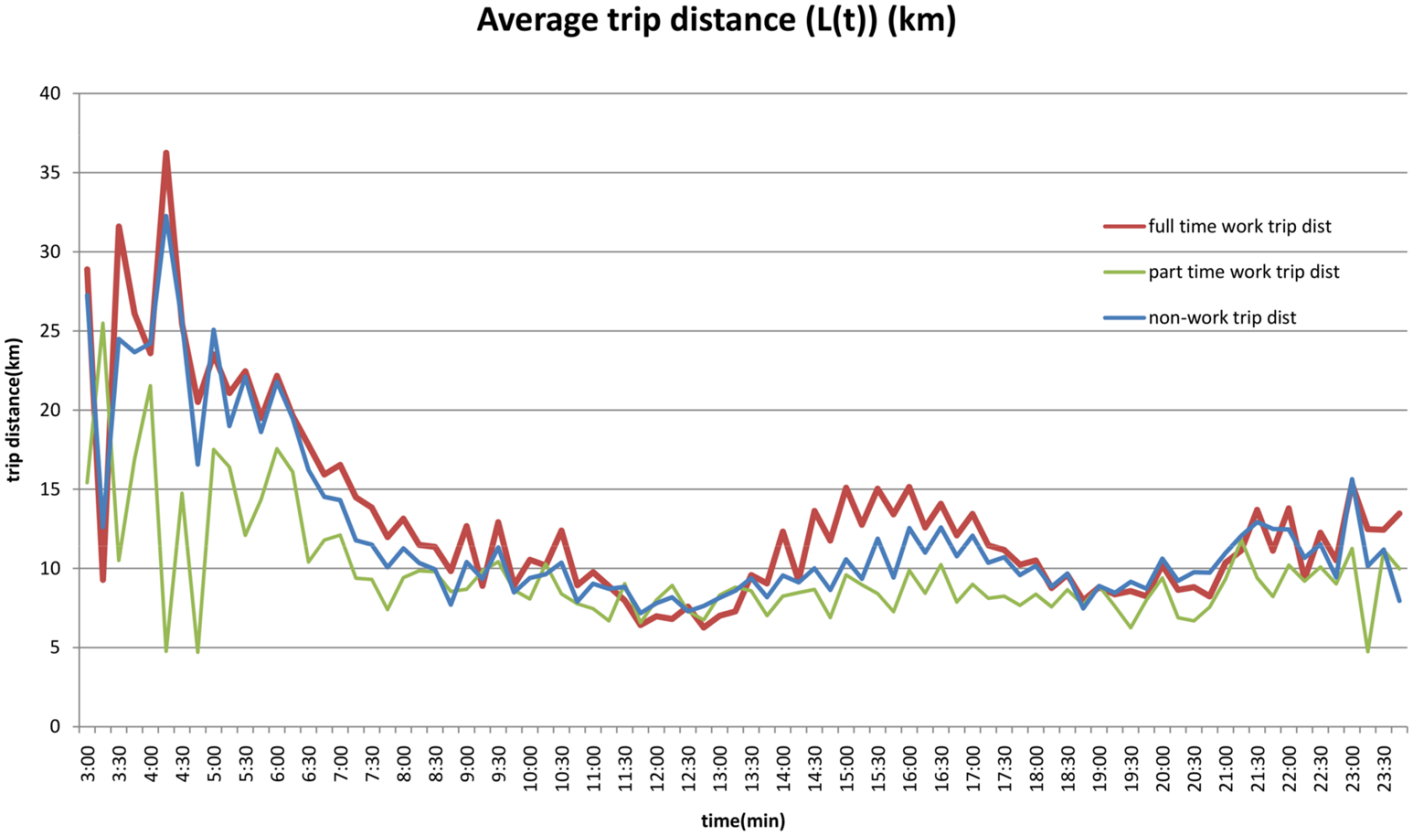
Average Trip Length by Time of Day and Purpose (2010).

Average network trip distance (on the shortest path route) by 15 minute period, in Fig 5, illustrates that full-time work trips are on average longer than part-time trips, and that trips in the early morning hours tends to be longer. People with long commutes depart earlier both to ensure on-time arrival, and to avoid congestion effects which are more onerous on longer distance trips.

**Fig 6:**
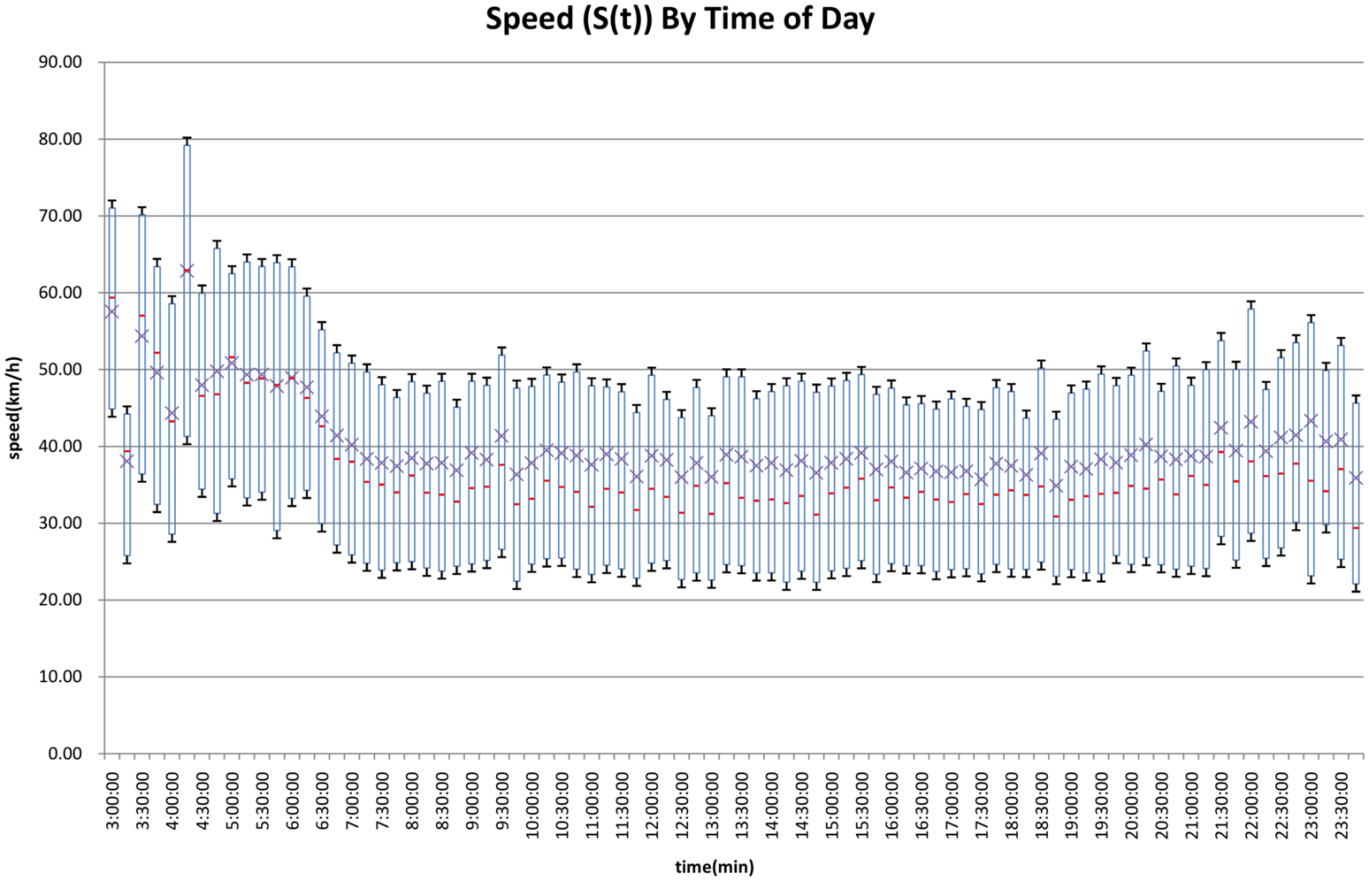
Average and Variance of Trip Speed by Time of Day (2010). Note: The figure represents mean with *x*, median as a bar, the box shows plus or minus one standard deviation, and the whiskers show 5th and 95th percentile speeds.

Fig 6 shows the change of (calculated) speed with time of day. The speed is quite high in the early morning and relatively flat the rest of the day, holding at 40km/h (probably closer to 80 km/h in actuality if we adjust for over-reporting of travel duration and under-estimation of distance). The median value of speed has a similar trend with average speed but lower values. The box plot diagram also show the distribution of speed at different time points, the deviation of speed is about 10km/h. The results show that in general, longer distance trips take more time. However we can see that there are differences by time of day, which we associate with (1) different congestion levels in different day parts, (2) different trip purposes in different day parts.

**Fig 7:**
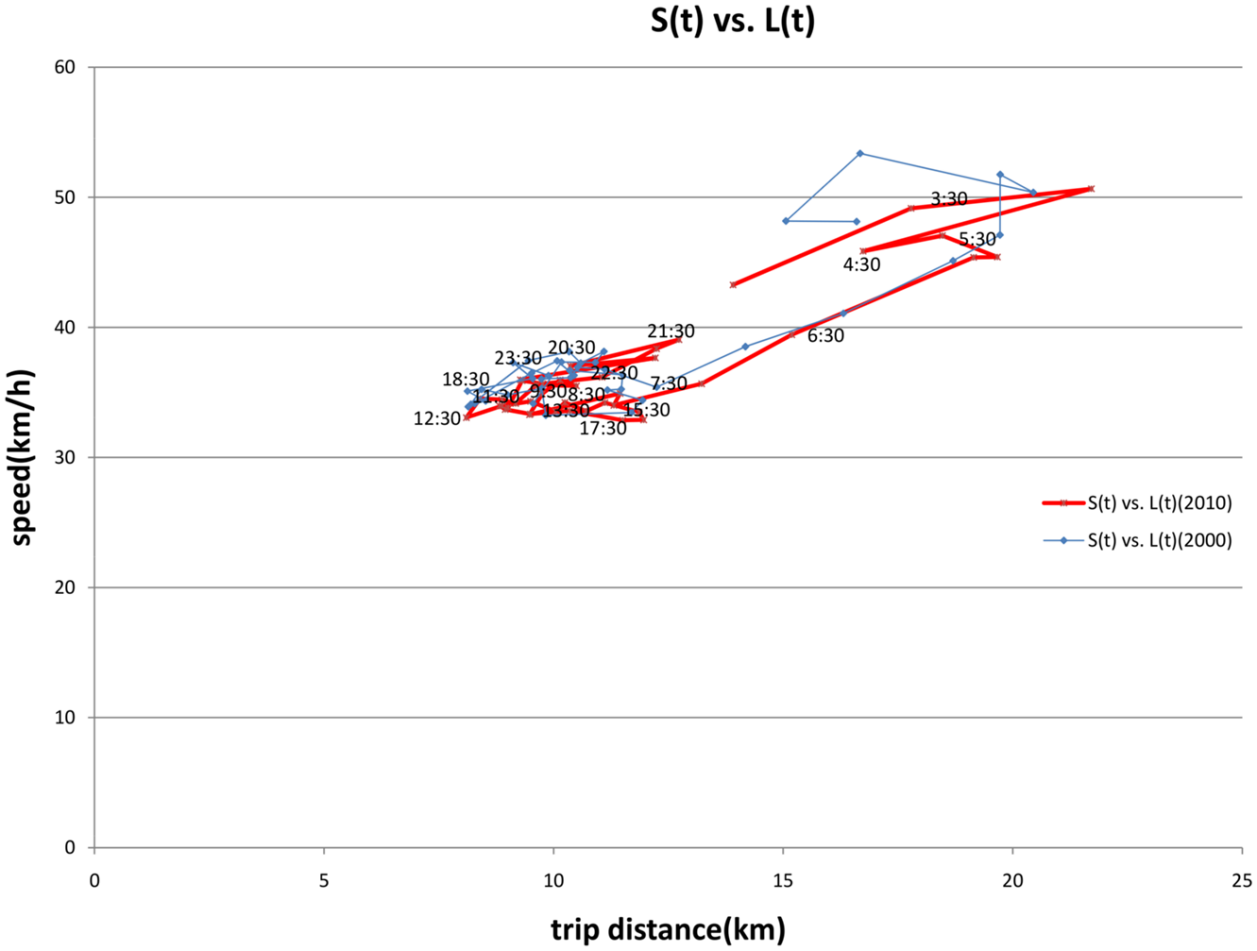
Relationship of Length and Speed (2000 and 2010).

The relationship of trip distance and speed is indicated in Fig 7. Long trips are associated with higher speeds, particularly for early morning departures, though the speeds drop noticeably after 7:00 am. From the ratio of speed to trip distance, congestion is greater in the afternoon than morning.

**Fig 8:**
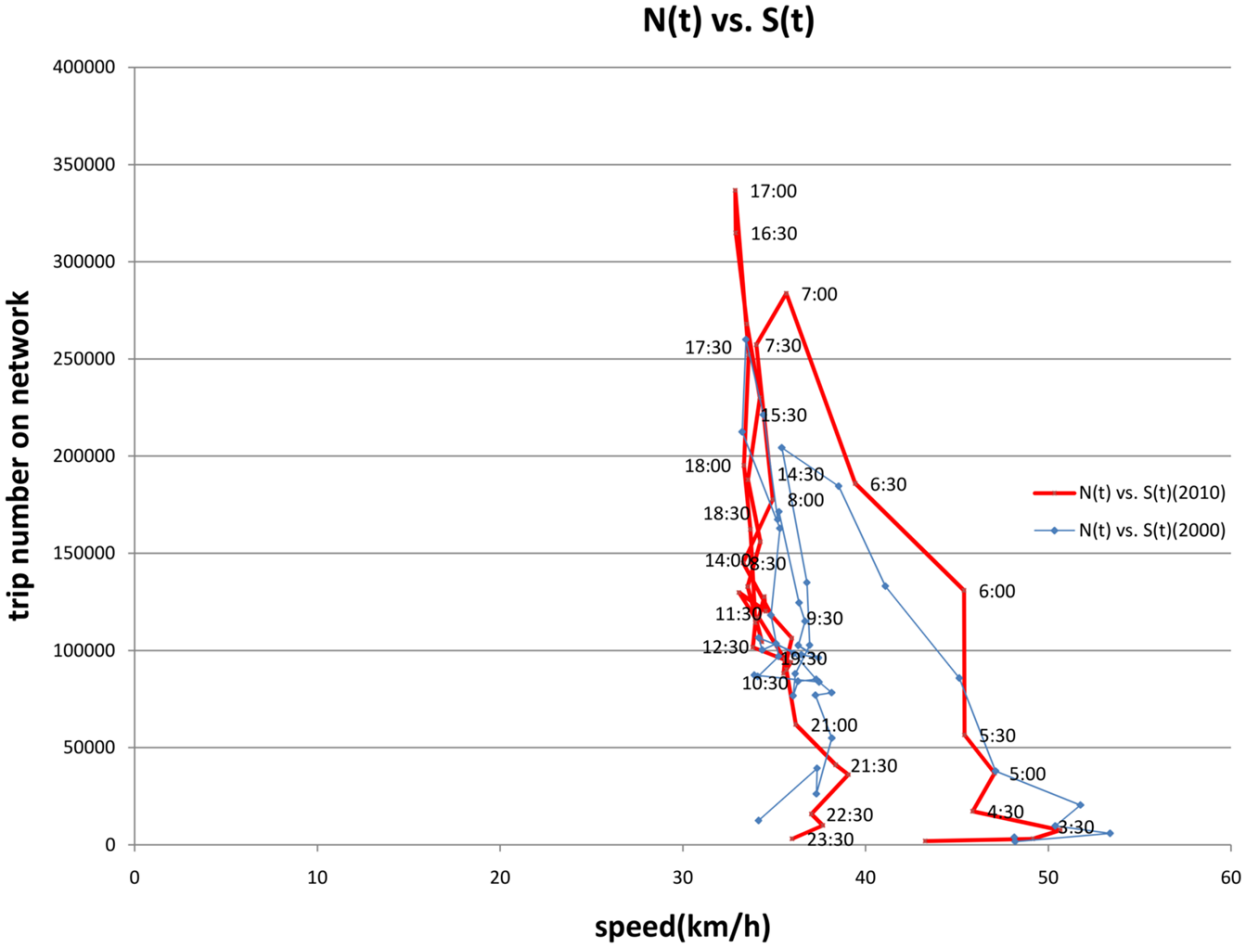
Fundamental Relationship of Speed and Number of Vehicles on the Network (2000 and 2010).

Fig 8 graphs the fundamental relationship in two-dimensions, and shows in general that as the more vehicles enter the network and thus traffic levels increase, speed decreases. But it also shows hysteresis type processes. There is not a single line, but rather 4 relationships: AM congestion onset (the left-most portion), Mid-day congestion off-set, PM congestion on-set, and PM congestion off-set. Unlike typical macroscopic or microscopic fundamental diagrams, we cannot assume trip length is fixed, making the relationship more complicated. So for instance, while one might expect with AM congestion off-set that speed rises, average network speed does not rise until the PM congestion on-set, and it continues rising with PM congestion off-set. What is going on (and we will illustrate in the next section) is that trip length is a confounding factor. The variability of trip length influences speed. We control for this to test hysteresis. Longer trips are faster, and are more likely to occur in the AM congestion onset and PM congested periods, while the shortest trips (typically mid-day nonwork trips) occur in less congested periods, but because they are shorter, and thus use more local roads, tend to have lower speeds.

**Fig 9:**
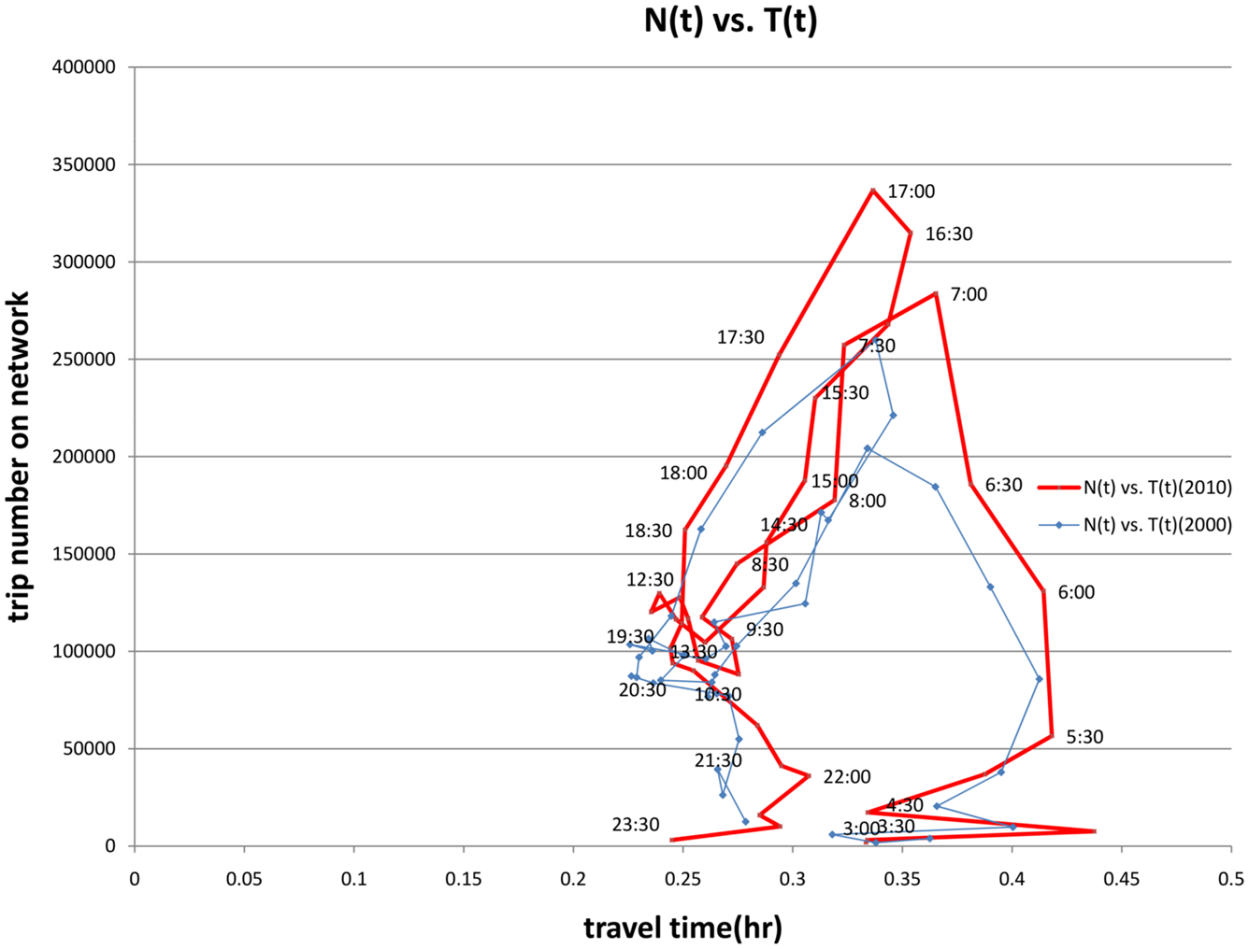
Travel Duration by Number of Vehicles on the Network (2000 and 2010).

That relationship becomes more obvious in Fig 9, showing the relationship between average travel duration and total vehicles on the network for every 15 minute period. The early morning has very long trips, which stabilize at about 24 minutes for the morning peak 15 minute period. Travel durations tend to decline through the mid-day as non-work (and part-time work) trips take a larger share of total travel. Durations then rise slightly in the afternoon peak, but do not match the morning, as the share of non-work trips is much higher.

### Statistical Results: Arrival and Departure Rates

A regression to determine the number of vehicles entering the network (*a*(*t*)) as a function of the number of vehicles on the network (*N*(*t*)) was estimated as a quadratic relationship.

The regression results are shown in Tables 2 and 3 for both the 2010 and 2000 TBI to test for temporal stability. The results vary by year. The results all show a positive relationship between *a*(*t*) and *N*(*t*), and a negative relationship with *N*(*t*)^2^, indicating a diminishing number of vehicles entering system as the traffic on the system rises. This resembles the relationships found with the macroscopic and microscopic fundamental diagrams. The difference between departure time and arrival time is trip duration, they both relate to a city’s dynamic state. Departure rate (exiting the network) depends on the state of traffic and arrival time. Arrival rate (entering the network) is the personal choice of each traveler, based on anticipated traffic levels (a function of historic traffic) and desired arrival (at destination) time.

**Table 2:** Dependent Variable: Arrival rate (*a*(*t*)) (2000 and 2010).

	AM				PM			
	2000							
	*B*	SE	t-stat	Sig.	*B*	SE	t-stat	Sig.
Constant	-204.832	3603.720	-0.057	0.955	-3843.335	7947.061	-0.484	0.631
*N*(*t*)	0.757	0.100	7.563	0.000	0.894	0.151	5.917	0.000
*N*(*t*)^2^	-7.621E-7	0.000	-1.228	0.228	-1.154E-6	0.000	-1.842	0.072
Sample Size	36 time periods				48 time periods			
Adj. *R*^2^	0.905				0.833			
	2010							
Constant	791.008	1659.744	-0.477	0.637	-1.689	2813.914	-0.001	1.000
*N*(*t*)	0.334	0.032	10.486	0.000	0.386	0.044	8.867	0.000
*N*(*t*)^2^	-2.917E-7	0.000	-2.469	0.019	-3.768E-7	0.000	-2.858	0.006
Sample Size	36 time periods				48 time periods			
Adj. *R*^2^	0.947				0.912			

**Table 3:** Dependent Variable: Departure rate (*d*(*t*)) (2000 and 2010).

	AM				PM			
	2000							
	*B*	SE	t-stat	Sig.	*B*	SE	t-stat	Sig.
Constant	-784.797	3088.386	-0.254	0.801	1265	2577	0.491	0.626
*N*(*t*)	0.367	0.059	6.191	0.000	0.406	0.040	10.183	0.000
*N*(*t*)^2^	-4.529E-7	0.000	-2.060	0.047	-5.111E-7	0.000	-4.233	0.000
Sample Size	36 time periods				48 time periods			
Adj. *R*^2^	0.831				0.913			
	2010							
Constant	-4643.379	4512.301	-1.029	0.311	494.159	716.040	0.069	0.945
*N*(*t*)	0.896	0.125	7.150	0.000	0.941	0.136	6.911	0.000
*N*(*t*)^2^	-1.796E-6	0.000	-2.311	0.027	-1.776E-6	0.000	-3.145	0.003
Sample Size	36 time periods				48 time periods			
Adj. *R*^2^	0.854				0.819			

In Table 4, the number of vehicles decreases faster (-35.814 vs. 24.111) in the morning peak hour while the vehicles increase faster (36.083 vs. -20.725) in the afternoon peak hour, so the number of vehicles increase fastest in the afternoon congestion onset and decrease fastest in the morning offset. This relates to the trip purpose of travelers. In the morning people need to get to their workplace on time, in the afternoon people get off work over a wider range of time and may engage in other activities.

**Table 4:** Dependent Variable: Number of Vehicles on the Network (*N*(*t*))—Comparing Onset and Offset of Congestion (2010).

	Onset				Offset			
	*B*	SE	t-stat	Sig.	*B*	SE	t-stat	Sig.
Morning								
Constant	-3.326E5	5.687E4	-5.848	0.000	1.370E6	1.146E5	11.957	0.000
*t*	24.111	3.069	7.858	0.000	-35.814	3.671	-9.756	0.000
Adj. *R*^2^	0.792				0.895			
Mid-day								
Constant	-3.166E5	7.742E4	-4.089	0.009	4.102E5	8.508E4	4.822	0.003
*t*	11.627	1.953	5.953	0.002	-5.425	1.834	-2.958	0.025
Adj. *R*^2^	0.852				0.525			
Afternoon								
Constant	-1.689E6	2.028E5	-8.329	0.000	1.681E6	1.165E5	14.423	0.000
*t*	36.083	3.689	9.782	0.000	-20.725	1.590	-13.036	0.000
Adj. *R*^2^	0.904				0.858			

### Statistical Results: Speed

Since we have a multi-dimensional relationship, a statistical model is employed to decompose the factors explaining average calculated travel speed by time of day, as shown in Table 5. We consider three factors: average (estimated shortest distance path) length of trip by time of day (*L*(*t*)), number of vehicles on the road at time t (*N*(*t*)), and a dummy variable indicating congestion onset (*c*). Other functional forms (e.g. quadratic) were tested without notable improvement in statistical performance.

$$\begin{array}{c} S(t)=f(L(t),N(t),c) \end{array}$$ (15)

where: *c* is a indicator [1, 0] variable for congestion onset. *c* = 1, if *N*(*t*) > *N*(*t* − 1). I.e. *N*(*t*) is increasing. This occurs in time periods: (3:00:00 - 7:00:00], (10:00:00 - 11:45:00], (13:45:00 - 16:30:00]). *c* = 0, otherwise. The congestion onset/offset variable is temporally aggregate, as small changes in *N*(*t*)*vs*.*N*(*t* − 1) are highly localized and not amenable to a metropolitan level analysis.

**Table 5:** Dependent Variable: Speed (2010 and 2000).

	Aggregate	(*S*(*t*))			Disaggregate	(*s*_*i*_)		
	*B*	SE	t-stat	Sig.	*B*	SE	t-stat	Sig.
	2000							
Constant	30.870	1.333	23.161	0.000	29.929	0.261	114.837	0.000
*L*(*t*), *l*_*i*_	0.001	0.000	8.582	0.000	0.001	0.000	112.870	0.000
*N*(*t*)	-8.826E-5	0.000	-6.509	0.000	-6.998E-5	0.000	-17.584	0.000
*c*	-1.196	0.724	1.652	0.103	-0.459	0.184	-2.495	0.013
Sample Size	84 time periods				30992 points			
Adj. *R*^2^	0.733				0.297			
	2010							
Constant	24.329	0.891	27.319	0.000	27.266	0.159	171	0.000
*L*(*t*), *l*_*i*_	1.279	0.074	17.274	0.000	0.957	0.578	153	0.000
*N*(*t*)	-1.898E-5	0.000	-7.456	0.000	-3.979E-5	0.000	-16.707	0.000
*c*	-0.773	0.532	-1.453	0.150	-0.843	0.156	-5.412	0.000
Sample Size	84 time periods				47685 points			
Adj. *R*^2^	0.869				0.334			

*t* indexes the time of day in 15 minute intervals t = [180–1440]

We put *L* (trip distance) into the model to predict speed (*S*(*t*), *s*_*i*_) because speed is not uniform, and depends on congestion and trip distance, and those are not fixed either.

The second regression results conduct for each individual trip, rather than by time slice, using each trip’s calculated trip length and calculated speed, rather than the averaged value by time of day.

$$\begin{array}{c} s_{i}=f\left(l_{i},N\left(t\right),c\right) \end{array}$$ (16)

As the number of observations rise, so does the variability between observations (the first regression averaged across multiple people traveling at a given time of day). Thus the adjusted *R*^2^ is lower (dropping from 0.87 in the aggregate model to 0.33 in the disaggregate model). Nevertheless the core relationships with Length (in this case individual trip length (*l*_*i*_)) and *N*(*t*) hold. All variables are significant at 5 percent level or better.

From the disaggregate model we see that each additional vehicle on the road in 2010 reduces network average speed by -0.00004 km/h. Each additional km of trip length increases network speed by 0.96 km. Moreover time periods where congestion is increasing (*c* = 1) reduce speed by 0.84 km/h. In 2010, length is a more important factor (based on the coefficient magnitudes) and number of vehicles on the network less important than in 2000. By Fig 1 we know that during congested periods, the cumulative number of vehicles entering and exiting the network is higher in 2010 than 2000, but for the whole day, there were slightly more vehicles on the network in 2000 than 2010.

To test hysteresis, Table 6 shows the coefficient *l*_*i*_ and *N*(*t*) for speed in morning peak hour, mid—day and afternoon peak hour using disaggregate data. The Hysteresis depends on variation in the number of vehicles on the network. For the speed regression, the constant for congestion onset is higher than that of offset in all six cases. The coefficient for length is higher in offset for 5 of 6 cases. Longer trips are more likely to be at the edge of the region, have faster speeds, and recover faster from congestion. The coefficient of number of vehicles on the network is higher (less negative) for offset than onset in 5 of 6 cases. Speed change is most sensitive to *l*_*i*_ and *N*(*t*) in the least congested mid-day period. Basically the coefficient of *l*_*i*_ is higher in the morning than afternoon and the coefficient of *N*(*t*) is lower in the morning than that in afternoon for the onset and this is opposite for the offset. The process by which speed is changes with number of vehicle and trip length is complicated. Still hysteresis can be found by the change of speed in different time periods and traffic levels.

**Table 6:** Dependent Variable: Speed—Comparing Onset and Offset of Congestion (2000 and 2010 TBI disaggregate data).

	Onset				Offset			
	2000							
	*B*	SE	t-stat	Sig.	*B*	SE	t-stat	Sig.
Morning								
Constant	35.384	0.795	44.520	0.000	27.787	0.812	34.210	0.000
*l*_*i*_	7.383E-4	2.21E-5	33.340	0.000	9.625E-4	2.47E-5	38.970	0.000
*N*(*t*)	-1.221E-4	1.17E-5	-10.42	0.000	-2.94E-5	1.36E-5	-2.170	0.030
Adj. *R*^2^	0.312				0.292			
Mid-day								
Constant	32.748	2.572	12.730	0.000	31.380	4.146	7.570	0.000
*l*_*i*_	1.096E-3	3.11E-5	35.271	0.000	1.122E-3	3.37E-5	33.320	0.000
*N*(*t*)	-1.715E-4	6.6E-5	-2.600	0.009	-1.479E-4	9.8E-5	-1.510	0.131
Adj. *R*^2^	0.281				0.294			
Afternoon								
Constant	30.760	0.710	43.330	0.000	28.845	0.355	81.270	0.000
*l*_*i*_	8.752E-4	1.78E-5	49.120	0.000	1.046E-3	1.43E-5	73.010	0.000
*N*(*t*)	-7.87E-5	1.09E-5	-7.210	0.000	-6.5E-5	5.17E-6	-12.570	0.000
Adj. *R*^2^	0.263				0.320			
	2010							
Morning								
Constant	29.815	0.587	50.810	0.000	26.583	0.359	73.990	0.000
*l*_*i*_	0.921	0.017	55.250	0.000	0.943	0.014	66.710	0.000
*N*(*t*)	-7.03E-5	6.51E-6	-10.800	0.000	-2.77E-5	5.38E-6	-5.140	0.000
Adj. *R*^2^	0.411				0.321			
Mid-day								
Constant	32.695	3.085	10.600	0.000	28.863	2.610	11.060	0.000
*l*_*i*_	1.129	0.026	42.760	0.000	1.093	0.024	45.810	0.000
*N*(*t*)	-2.236E-4	8.39E-5	-2.670	0.008	-1.4E-4	7.34E-5	-1.900	0.057
Adj. *R*^2^	0.305				0.340			
Afternoon								
Constant	27.235	0.395	69.040	0.000	26.675	0.295	90.300	0.000
*l*_*i*_	0.851	0.012	73.420	0.000	1.012	0.013	80.300	0.000
*N*(*t*)	-4.01E-5	4.36E-6	-9.190	0.000	-3.454E-5	3.95E-6	-8.750	0.000
Adj. *R*^2^	0.300				0.346			

## Discussion

This analysis uses a travel survey to measure total traffic levels and finds statistically significant relationships between aggregate demand and individual and average travel speed. we also find a hysteresis process, whereby speed depends not only on vehicles entering or exiting the network and number of vehicles on the network, but also on whether congestion is rising or falling. A strength of this analysis is the ability to use historic travel surveys to construct these curves for previous years where traffic speed data may not have been collected. Travel surveys have been collected systematically since the 1940s, and a number of those from the 1960s and 1970s still survive (many are archived at the Metropolitan Travel Survey Archive http://surveyarchive.org). While not every survey has the spatial detail or weighting scheme we might like today, the general results should be comparable, and can show the stability (or lack thereof) in metropolitan speed, flow, length relationships.

Cautions about the analysis include that this is based solely on personal travel reported in the survey, and this excludes non-personal trucks, buses, bicycles and other vehicles on the road. However even with such limits, the relationships between speed, trip distance, and trip duration are sufficiently stable that strong fundamental relationships can be observed using travel survey data.
